# The Durbar Honours

**Published:** 1911-12-16

**Authors:** 


### The Durbar Honours.

### DECORATIONS FOR THE INDIAN MEDICAL SERVICE.

ON Monday night the list of Durbar Honours and appointments in the Indian Orders was issued as a supplement to the *London Gazette*. It contains many names distinguished in the hospital and medical world of India:

**K.C.S.I.** — Surg.-Gen. Charles P. Lukis, C.S.I., M.B., F.R.C.S., Director-General Indian Medical Service, and an Additional Member of the Council of the Governor-General of India for making Laws and Regulations. Sir C. P. Lukis, who studied at Bart's, was formerly Principal and Professor of Medicine at Medical College, Calcutta, and first physician, College Hospital.

Surgeon-General Francis Wollaston Trevor, C.B., M.B., K.H.S., Principal Medical Officer, India, and Hon. Surgeon to the King. Sir F. W. Trevor studied at Guy's and Aberdeen University, has held various appointments in the I.M.S., and served in the Afghanistan, Soudan, and South African wars, for which he has received medals.

**C.S.I.**—Lieut.-Colonel William Burney Bannerman, M.D., I.M.S., Surgeon-General with the Government of Madras, and lately Director Bombay Bacteriological Laboratory.

Colonel George Francis Angelo Harris, M.D., V.H.S., I.M.S., Inspector-General of Civil Hospitals, Bengal.

Lieutenant-Colonel Arthur Russell Aldridge, R.A.M.C., lately Sanitary Officer, Army Headquarters.

**K.C.I.E.** — Surg.-Gen. Arthur Mudge Branfoot, C.I.E., F.R.C.S., M.B., I.M.S. (retired), President of the Medical Board, India Office. Sir A. M. Branfoot studied at Guy's and London University, and entered the I.M.S. in 1872, retiring in 1903.

**C.I.E.** — Major Leonard Rogers, M.D., I.M.S., Professor of Pathology, Medical College, Calcutta, and Bacteriologist to the Government of India.

Lieutenant - Colonel James Reed Roberts, I.M.S., Residency Surgeon in Indore and Administrative Medical Officer in Central India.

Major Frederick Fenn Elwes, M.D., I.M.S., Surgeon to the Governor of Madras.

**C.V.O.** — Colonel Charles J. Bamber, I.M.S., Inspector-General of Civil Hospitals, Punjab, and a Member of the Coronation Durbar Committee.

**Knight Bachelor.** — Lieutenant-Colonel Charles Henry Bedford, M.D., D.Sc., I.M.S., Chemical Examiner, Bengal.

**Kaisar-i-Hind.**—The following have been awarded the "Kaisar-i-Hind Medal for Public Service in India" of the First Class:

Major Albert Elijah Walter, I.M.S., Superintendent *x*-Ray Institute, Dehra Dun.

Major A. Gwyther, M.B., F.R.C.S., I.M.S., Civil Surgeon, Howrah, and Supt. of the Howrah Gaol.

Rai Hari Mohan Chandra Bahadur, Secretary, Lowis Jubilee Sanitarium, Darjeeling.

Dr. Thomas Joseph O'Donnel, lately Chief Medical Officer, Kolar Gold Fields.

Captain Jasper Robert Joly Tyrrell, M.B., I.M.S., Agency Surgeon, Bhopawar, Central India.

Major William Hancock Tucker. M.R.C.S., L.R.C.P., I.M.S., District Medical and Sanitary Officer, Coimbatore, Madras Presidency.

Dr. Herbert F. Lechmere Taylor, M.B., Ph.D., M.A., of the Church of Scotland Mission, Jalalpur, and in charge of the Jalalpur Hospital.

The Rev. J. C. Young, Medical Missionary of the Keith Falconer Mission at Sheikh Othman, Aden.

Dr. Raghavendra Row, M.D., D.Sc., of Bombay.

